# Quality Evaluation and Multi-Criteria Optimization of Cookies Fortified with Lyophilized Black Goji

**DOI:** 10.3390/foods15101733

**Published:** 2026-05-14

**Authors:** Katarina Šavikin, Gordana Zdunić, Jelena Živković, Nada Ćujić Nikolić, Dejan Gođevac, Milica Nićetin, Jelena Filipović, Vladimir Filipović

**Affiliations:** 1 Institute for Medicinal Plants Research, Dr Josif Pančić, Tadeuša Košćuška 1, 11000 Belgrade, Serbia; ksavikin@mocbilja.rs (K.Š.); gzdunic@mocbilja.rs (G.Z.); jzivkovic@mocbilja.rs (J.Ž.); ncujic@mocbilja.rs (N.Ć.N.); 2 Institute of Chemistry, Technology and Metallurgy, University of Belgrade, Studentski trg 12-16, 11000 Belgrade, Serbia; dejan.godjevac@ihtm.bg.ac.rs; 3 Faculty of Technology Novi Sad, University of Novi Sad, Bul. Cara Lazara 1, 21000 Novi Sad, Serbia; milican@uns.ac.rs; 4 Institute of Food Technology, University of Novi Sad, Bul. Cara Lazara 1, 21000 Novi Sad, Serbia; jelena.filipovic@fins.uns.ac.rs

**Keywords:** *Lycium ruthenicum*, confectionary product, technical quality, anthocyanins, antidiabetic

## Abstract

Lyophilized black goji berry powder (LBGBP) cultivated in Serbia was evaluated and optimized as a fortification agent in cookie formulation. Nutritional, chemical, technical and biological characteristics, in vitro release and storage stability were analyzed. LBGBP is characterized by a phenolic-rich profile dominated by acylated anthocyanins, 5-*O*-caffeoylquinic acid (5-*O*-CA), and spermidine-based phenylamides (S1, S2), which are partially retained in LBGBP-enriched cookies and enhance their functional properties. The substitution of different white flour shares with LBGBP in cookies statistically significantly improved their overall nutritional profile by increasing protein, dietary fiber, minerals and bioactive compounds, concurrently reducing fat, sugar and sodium levels. With the increase in the LBGBP in cookies, total phenolics and total anthocyanin content increased to the levels of 58.09 mg GAE/100 g and 10.12 mg CGE/100 g of cookies, respectively. The overall effect of LBGBP cookie fortification led to softer, more crumbly cookies with significant improvement in antioxidant and antidiabetic activity. The Z-score analysis was chosen to perform multi-criteria cookie formulation optimization with the goal of maximal functional enrichment, with minimal decrease in technological quality. The 10% LBGBP substitution was calculated to produce optimal overall quality, obtaining 65.96% of maximal score in comparison to the control sample of only 32.91%.

## 1. Introduction

Plants from the genus *Lycium* (*Solanaceae*) are distributed in subtropical and temperate climatic regions and encompass approximately 80 species, some of which are known as goji [1,2]. Their medicinal and nutritional value is widely recognized, largely due to their rich and diverse phytochemical composition, particularly phenolic compounds, polysaccharides, terpenes, and lipids [3,4]. The most valued medicinal species, *Lycium barbarum L.* and *L. chinense Miller*, known as red goji berries, have higher amounts of carotenoids, while *L. ruthenicum Murray*, commonly known as black goji, has a predominant content of anthocyanins known as potent antioxidants [2]. Traditionally, especially in Chinese and Tibetan medicine, goji is used to treat headaches, fatigue, infertility, and as an anti-aging agent, and it can also be incorporated into various food products [1,5]. These traditional applications have been increasingly supported by modern scientific research. Numerous clinical studies have proven its beneficial properties including neuroprotective, anticancer, hepatoprotective, anti-inflammatory, anti-obesity, antidiabetic, and antioxidant [6,7]. However, these studies are based on extracts or purified compounds administered at relatively high concentrations, which may not directly reflect the effects observed when goji berries are incorporated into complex food matrices. Increasing interest in cultivation has contributed to the intensive cultivation of *Lycium* species worldwide [8].

In the food industry, *Lycium* berries are used both as a dried fruit intended for direct consumption, as well as integrated into various products [1,4]. A good quality of dried berries is achieved using modern drying technologies and proper pretreatments, providing satisfactory sensory properties and nutritional value [9]. Goji berry has high water and sugar content, a thin and waxy covered epidermis, and a dense fleshy structure of cells, making the drying process more demanding. Among the different drying methods applicable to food processing industries, one of the most advanced is freeze drying, especially for the drying of products and ingredients sensitive to heat [10]. Lyophilization enables superior preservation of phenols, polysaccharides, and anthocyanins, while maintaining desirable sensory and rehydration properties of goji berries [1,9]. Due to their high number of flavor varieties, readiness to eat, long shelf life, and reasonable price, cookies are among the top-rated wheat-based products that are consumed worldwide. The addition of ingredients rich in biologically active compounds opens up the possibility of significant improvements in their nutritional and functional properties [11,12]. On the other hand, every ingredient in a complex system, such as a cookie, has an essential purpose, and changes to the standard formulation can lead to dough changes, affecting the final product’s quality [13]. Due to this, quality and production optimization, as well as precise testing, are required in the cookie formulation with the addition of new ingredients [12].

Despite the well-documented benefits of black goji berries, their application in baked goods remains underexplored, particularly regarding the retention of bioactive compounds during thermal processing and the optimization of substitution levels for consumer acceptance. Hence, our study aimed to perform a chemical characterization of black goji berries (lyophilized powder—LBGBP) grown in Serbia, a region with different climatic and agroecological characteristics compared to their natural habitat, to formulate a cookie with the addition of LBGBP and to define the cookies’ nutritional, chemical, and sensory characteristics. In order to examine the potential benefits on human health, the antioxidant potential and antidiabetic properties were also examined in berries and cookies. Finally, the effect of storing the cookies in different conditions was also examined in order to determine the optimal one.

## 2. Materials and Methods

### 2.1. Materials

#### 2.1.1. Black Goji Powder

Lyophilized black goji berry powder (LBGBP) was obtained from the fresh goji berries, collected in September 2024 from the plantation established by family agricultural enterprise “Plantaža Marjanović” located on the borders of Pančevo, Serbia, along the road to the village of Novo Selo at an altitude of approximately 77 m a.s.l. The entire cultivation process adheres to organic production standards. After the harvest process, the berry fruits were initially frozen in commercial freezers at −18 °C. Further, they underwent a freeze-drying process (lyophilization) (Model LF 100) at Elbi d.o.o., Valjevo, which included the following stages: rapid freezing where the fruits were rapidly frozen at −60 °C within a few hours to stabilize internal cellular structures and prevent the formation of large ice crystals; primary drying, where under controlled vacuum conditions frozen water was removed from the berries through sublimation process. This phase lasted approximately 24–36 h, during which about 90–95% of the moisture was removed. Secondary drying (desorption phase) was performed to eliminate residual bound water within the final drying phase, conducted over approximately 72 h, reducing the final moisture content to below 5%.

Further, fruits were ground into a fine powder (MILL130, Bakery Pastry and Pizza Equipment, RAM S.r.l., Vicenza, Italy) and sieved using a set of sieves with mesh sizes of 0.30 mm and 0.15 mm (Yugoslavian Pharmacopeia IV) to obtain a fraction between 150 and 300 µm. The selected fraction was then packaged in triplex barrier pouches filled with inert nitrogen gas (Besser Vacuum S.r.l., Dignano, Italy).

#### 2.1.2. Materials for Cookie Preparation

The following materials used for the production of cookie samples was obtained from local sources: white wheat flour, type: T-400, moisture content: 14% (“Danubius”, Novi Sad, Serbia); margarine (“AD Dijamant”, Zrenjanin, Serbia); sugar (“Šajkaška” Žabalj, Serbia); NaCl (“Produkt”, Stara Pazova, Serbia); NaHCO3 (“Aleva”, Novi Kneževac, Serbia); and glucose (“BIO-UNA”, Novi Sad, Serbia).

### 2.2. Cookie Sample Production

Cookie samples without (C0) and with LBGBP (C2.5, C5, C7.5 and C10) flour substitution were produced in the Institute of Food Technology in Novi Sad, in their pilot plant for bakery products, following appropriate AACC methods [14]. The main production operations included dough mixing, processing, and baking processes. An experimental design plan that integrates different quantities of LBGBP flour substitution (from 0 to 10% in 2.5% increments) is presented in Supplementary Materials, Table S1.

**Figure 1:**
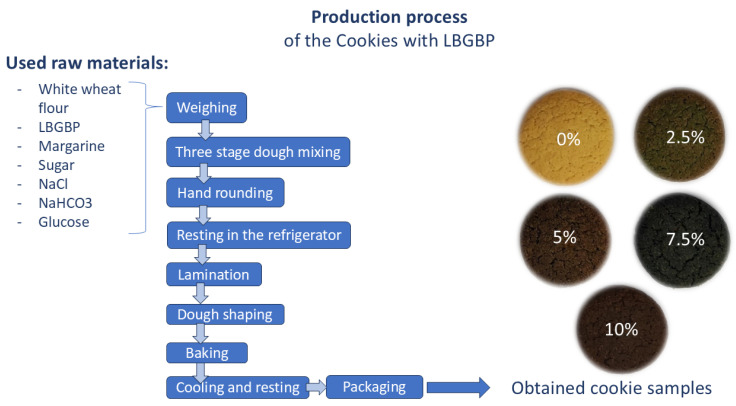
Production process of the cookie samples without and with LBGBP flour substitution.

The dough mixing, handling and baking procedure is described in more detail in Filipović et al. [12] and it can also be seen in Figure 1.

### 2.3. Methods of Chemical Content Determination

#### 2.3.1. Methods of Mineral Matter Content Determination

The mineral composition of LBGBP and cookies, both without and with LBGBP flour substitution, was determined using AOAC standard methods: zinc—Zn, copper—Cu, iron—Fe, potassium—K, magnesium—Mg, calcium—Ca, manganese—Mn and sodium—Na. Mineral composition was determined via atomic absorption spectrophotometry, method No. 984.27 using Varian Spectra AA 10 (Varian Techtron Pty Ltd., Mulgrave, Victoria, Australia) apparatus. Each test was done in triplicate.

#### 2.3.2. Methods of Food Nutrient Content Determination

Proximate food nutrient content of LBGBP and cookies without and with LBGBP flour substitution was done according to AOAC standard methods [15]: moisture content—method No. 926.5, protein content—method No. 950.36, fat content—method No. 935.38, sugar content—method No. 80-68, cellulose content—method No. 973.18, ash content—method No. 930.22, and total carbohydrate content—method No. 2020.07. Each test was done in triplicate.

#### 2.3.3. Determination of the Total Phenolics and Total Anthocyanin Content

For the determination of TPC in samples, the Folin–Ciocalteu (FC) spectrophotometric method was utilized [16]. Approximately 500 mg of LBGBP and 5 g of cookies were separately extracted in an ultrasonic bath for 30 min using 5 mL and 10 mL of MeOH, respectively. Extracts (100 µL) were mixed with FC reagent (diluted with distilled water 1:10, *v*/*v*; 750 µL). After incubation for 5 min, sodium carbonate (Na_2_CO_3_) solution (60 g/L; 750 µL) was added. In order to promote color development, the incubation was continued for 90 min in the dark at room temperature. The solution was filtered using PTFE membrane filter, and the absorbance was measured at 725 nm. The experiment was performed in triplicate and results expressed as milligrams of gallic acid equivalents per 100 g of sample (mg GAE/100 g).

For determination of the TAC in samples, the procedure according to Đorđević et al., [17], with slight adaptation, was utilized. A 50-fold dilution of sample extracts was prepared in 0.1% HCl in methanol. The absorbance was measured at 528 nm. A compensating solution was 0.1% HCl in methanol. The experiment was performed in triplicate, and results expressed as milligrams of cyanidin-3-*O*-glucoside equivalent per 100 g of sample (mg CGE/100 g). The percentage content of anthocyanins was calculated from the formula:

(1) $$Content\left(\%\right)=A\cdot \frac{5000}{718}\cdot m\cdot 100$$

where *A* is the absorbance at 528 nm, 718 is the specific absorbance of cyanidin-3-*O*-glucoside chloride at 528 nm, and *m* is the mass of the examined substance in grams.

#### 2.3.4. LC/MS Analysis

The chemical profiles of LBGBP and cookie samples (C0–C10) were characterized by liquid chromatography coupled to high-resolution mass spectrometry using a Thermo Scientific Vanquish Core HPLC system hyphenated to an Orbitrap Exploris 120 mass spectrometer (San Jose, CA, USA). Chromatographic separation was carried out on a Lichrospher RP-18 column (250 × 4 mm, 5 μm) thermostatted at 25 °C. The mobile phase consisted of solvent A (water with 1% formic acid, *v*/*v*) and solvent B ([acetonitrile or methanol] containing 1% formic acid, *v*/*v*). The flow rate was set to 0.5 mL/min and the injection volume was 5 μL. The gradient elution program was as follows: 5–18% B from 0 to 10 min, 18–35% B from 10 to 35 min, 35–100% B from 35 to 40 min, isocratic at 100% B from 40 to 42 min, return to 5% B from 42 to 43 min, and re-equilibration at 5% B until 50 min.

The Orbitrap Exploris 120 was equipped with an electrospray ionization (ESI) source operated in negative and positive ion mode. Full-scan mass spectra were recorded over an *m*/*z* range of 100–1500 at a resolving power of 60,000 (FWHM at *m*/*z* 200). Data-dependent MS/MS (dd-MS2) experiments were triggered for the most intense precursor ions, which were isolated and fragmented using a normalized collision energy of 35%, with an Orbitrap resolving power of 15,000. Dynamic exclusion was enabled (exclusion duration 10 s, maximum of two repeated selections per precursor), and the intensity threshold for MS/MS acquisition was set at 1 × 10^5^. Instrument control and data acquisition were performed with Xcalibur software, version 4.2 (Thermo Fisher Scientific, Waltham, MA, USA) [18].

#### 2.3.5. HPLC Analysis

Quantitative determination of the dominant phenolic compounds in LBGBP and cookies (C0–C10) was performed by high-performance liquid chromatography with diode-array detection (HPLC–DAD) using an Agilent 1260 Infinity II system (Agilent Technologies, Waldbronn, Germany) equipped with a binary pump, autosampler, column oven and DAD detector. Chromatographic conditions (stationary phase, mobile phases, gradient program, flow rate, column temperature and injection volume) were the same as those described for the LC–MS analysis in Section 2.3.4. Detection was carried out at 320 nm for hydroxycinnamic acid and 530 nm for anthocyanins.

External calibration curves were constructed using authentic standards of 5-*O*-caffeoylquinic acid (≥98%, Extrasynthese, Genay, France) and petunidin 3-*O*-glucoside chloride (≥98%, ChemFaces, Wuhan, China). Stock solutions of each standard were prepared in methanol and diluted to obtain a series of working solutions at different concentration levels, which were injected in triplicate. Calibration curves were obtained by plotting peak area versus concentration.

The concentration of 5-*O*-caffeoylquinic acid in LBGBP and cookie samples was determined directly from its own calibration curve. Three acylated petunidin derivatives—petunidin-3-*O*-(glucosyl-*trans-p*-coumaroyl)-rutinoside-5-*O*-glucoside, petunidin-3-*O*-(caffeoyl)-rutinoside-5-*O*-glucoside, and petunidin-3-*O*-(*trans-p*-coumaroyl)-rutinoside-5-*O*-glucoside—were quantified using the calibration curve of petunidin 3-*O*-glucoside chloride and their contents are expressed as petunidin 3-*O*-glucoside equivalents. All analyses were performed in triplicate, and results are reported as mean values ± standard deviation.

### 2.4. Methods of Cookie Samples’ Technical Quality Characteristics Analysis

The technological quality characteristics of the cookie samples were analyzed according to the AACC 10–50D method [14].

Determination of the baking weight loss (*BWL*) was done by weighing cookie samples before and after baking and calculating according to the equation:

(2) $$BWL\;\left(\%\right)=\frac{\left(m_{0}-m_{t}\right)}{m_{0}}\cdot 100$$

where *m*_0_ is the cookie sample’s weight before baking (g) and *m_t_* is the cookie’s weight after baking (g). Analysis was performed on six samples.

Drying weight loss (*DWL*) was determined by measuring the weight of cookies after the baking stage and after 30 min of cooling at room temperature, using the following equation:

(3) $$DWL\;\left(\%\right)=\frac{\left({m_{0}}^{\prime }-{m_{t}}^{\prime }\right)}{{m_{0}}^{\prime }}\cdot 100$$

where *m*_0_′ is cookie weight after baking (g) and *m_t_*′ is cookie weight after 30 min of cooling (g). Analysis was performed on six samples.

Cookie sample dimensions were determined after a cooling period of 30 min. Cookie sample diameter measurements in the lamination direction (length—L), normal to the lamination direction (width—W), and the cookie samples’ thickness (T), were recorded.

The average of cookie samples (R) was determined by the lowest W and highest L values.

The cookie samples’ T values were measured by placing six cookies in a column and determining their total height. After first determination, six cookie samples were rearranged in the column and their height was determined again. At the end, the obtained median value was divided by the number of cookies, 6, to calculate the median value of the cookie samples—T.

Diameter-to-thickness ratio (R/T) was calculated by dividing the obtained values of R and T for every individual sample tested. This ratio indicates the cookie samples’ shape deformation during baking. All measurements were done in triplicate. Results are expressed as mean value ± standard deviation.

### 2.5. Methods of Cookie Samples’ Texture Characteristics Instrumental Analysis

A texture analyzer, TA-XT2 (Stable Micro System, Godalming, UK), containing a 25 kg load cell and Knife Edge with Shotted Insert HPD/bs tools was used for determination of the cookie samples’ texture characteristics. Analysis was performed by using compression mode at the crosshead speed of 1 mm/s before, 3 mm/s during, and 10 mm/s after the analysis. Using computer software (Exponent Stable MicroSystems, version 6.0) the following responses were obtained: maximum force (N) and distance at break, in the function of time, which represents indicators of the cookies’ hardness. Analyses of the cookie samples’ textural characteristics were done in six repetitions for every cookie batch, 24 h post-baking, at a room temperature of approximately 25 °C.

### 2.6. Methods of Cookie Color Characteristics Instrumental Analysis

Cookie samples’ surface color parameters were determined 24 h after baking, by application of a chroma meter (CR-400, Konica, Minolta, Tokyo, Japan)—a tri-stimulus colorimeter, as explained in detail in Filipović et al. [19] and Košutić [20].

Testing of all cookie samples’ surface color parameters was done in six repetitions.

### 2.7. Storage Stability

Cookies (C0–C10) were used for the stability test analysis. Three separate clear-capped glass bottles with investigated samples were individually stored for 30 days at 4, 25, and 40 °C, in a dark environment, simultaneously. Storage stability under varying temperature ranges was assessed by testing TPC, TAC and dominant phenolic compounds.

### 2.8. Methods of Biological Activity Testing

#### 2.8.1. DPPH Radical Scavenging Activity

The capability of LBGBP and cookies without and with LBGBP flour substitution to scavenge 2,2-diphenyl-1-picrylhydrazyl (DPPH·) radicals was estimated following the method of Konić-Ristić et al. [21].

#### 2.8.2. Antidiabetic Activities

##### Inhibition of α-Glucosidase

The α-glucosidase inhibitory capability of LBGBP and cookies C0 and C10 was estimated following the method of Radan et al. [22]. The absorbances were measured at 400 nm against a blank. The percentage inhibition of enzyme activity was calculated using the formula:

(4) $$\%\;Inhibition=\left[\frac{\left(A_{c}-A_{s}\right)}{A_{c}}\right]\cdot 100$$

where *A_c_* is the absorbance of the control (the extracts were replaced with phosphate buffer), and *A_s_* is the absorbance of the extracts. The results are expressed as IC_50_ values (µg/mL). Acarbose was used as a positive control.

##### Inhibition of α-Amylase

The α-Amylase inhibitory capability of LBGBP and cookies C0 and C10 was estimated according to the method of Radan et al. [22]. The absorbances were measured at 540 nm against a blank. The percentage inhibition of enzyme activity was calculated using the formula:

(5) $$\%\;Inhibition=\left[\frac{\left({A_{c}}^{\prime }-{A_{s}}^{\prime }\right)}{{A_{c}}^{\prime }}\right]\cdot 100$$

where *A_c_*′ is the absorbance of the control (the extracts were replaced with phosphate buffer), and *A_s_*′ is the absorbance of the extracts. The results are expressed as IC_50_ values (µg/mL). Acarbose was used as a positive control.

### 2.9. In Vitro Release Study

The C10 sample was used to investigate the release profiles of dominant phenolic compounds (5-*O*-caffeoylquinic acid; petunidin-3-*O*-(glucosyl-*trans-p*-coumaroyl)-rutinoside-5-*O*-glucoside; and petunidin-3-*O*-(*trans-p*-coumaroyl)-rutinoside-5-*O*-glucoside) at pH 1.2 (0.1 M HCl) and 6.8 (phosphate buffer) using conditions reported in the 10th European Pharmacopoeia [23], with slight modifications. Cookies were chopped before the analysis in order to mimic cookie mastication in the mouth. The study was conducted in beakers placed in the Erweka DT70, Erweka, Langen, Germany, water bath at 100 rpm for a period of 120 min at 37 ± 1 °C. Accurately weighed samples (three replications, approximately 5 g) were tested in 20 mL of prepared dissolution medium. Small volumes of medium (2 mL) were drawn at fixed time intervals (0, 15, 30, 60, 120 min), filtrated and immediately replaced with 2 mL of fresh medium. The procedure for botanical dosage forms described at the United States Pharmacopeial Convention [23] was used to prepare the pooled sample while HPLC (Section 2.3.5) was used to determine the concentrations of marker compounds in the collected samples.

### 2.10. Statistical Analysis

Statistical data processing was performed using SPSS version 29, Trial software (Dublin, Ireland). The results are expressed as mean value ± standard deviation. One-way ANOVA (95% confidence level) followed by Tukey’s post hoc test was employed to determine statistically significant differences (*p*-values lower than 0.05) among the extraction abilities of the target compounds.

#### Z-Score Analysis

Z-score analysis was applied in the pursuit of mathematical optimization, converting cookie samples’ quality responses, via min–max normalization, into a dimensionless, comparable scale. In this manner direct comparison and further statistical evaluation between different samples was allowed [24]. The highest obtained total Z-score value, calculated from the combination of all segment Z-scores, indicates the optimal total or overall quality profile of the cookie samples [25].

Segment Z-scores are calculated as follows:

Cookie samples’ chemical composition:

(6) $$S_{1i}=\frac{\sum_{k=1}^{4}\left(\frac{x_{ki}-x_{kmin}}{x_{kmax}-x_{kmin}}\right)+\sum_{j=1}^{3}\left(1-\frac{x_{ji}-x_{jmin}}{x_{jmax}-x_{jmin}}\right)}{7}$$

where *x_k_* are: moisture, proteins, cellulose and ash composition; and *x_j_* are: fat, sugars and total carbohydrate composition.

Cookie samples’ mineral matter composition:

(7) $$S_{2i}=\frac{\sum_{l=1}^{6}\left(\frac{x_{li}-x_{lmin}}{x_{lmax}-x_{lmin}}\right)+\left(1-\frac{x_{mi}-x_{mmin}}{x_{mmax}-x_{mmin}}\right)}{7}$$

where *x_l_* are: Zn, Fe, K, Mg, Ca, and Mn; and *x_m_* is Na.

Cookie samples’ total phenols and anthocyanin content:

(8) $$S_{3i}=\frac{\sum_{n=1}^{2}\left(\frac{x_{ni}-x_{nmin}}{x_{nmax}-x_{nmin}}\right)}{2}$$

where *x_n_* are TCA and TAC.

Cookie samples’ technical quality:

(9) $$S_{4i}=\frac{\sum_{o=1}^{4}\left(1-\frac{x_{oi}-x_{omin}}{x_{omax}-x_{omin}}\right)+\left(\frac{x_{pi}-x_{pmin}}{x_{pmax}-x_{pmin}}\right)}{5}$$

where *x_k_* are: *BWL*, *DWL*, R and R/T; and *x_p_* is T.

Cookie samples’ texture instrumental analysis:

(10) $$S_{5i}=\frac{\sum_{q=1}^{2}\left(1-\frac{x_{qi}-x_{qmin}}{x_{qmax}-x_{qmin}}\right)}{2}$$

where *x_q_* are hardness and fracturability.

Cookie samples’ color instrumental analysis:

(11) $$S_{6i}=\frac{\sum_{r=1}^{3}\left(\frac{x_{ri}-x_{rmin}}{x_{rmax}-x_{rmin}}\right)+\left(1-\frac{x_{si}-x_{smin}}{x_{smax}-x_{smin}}\right)}{4}$$

where *x_r_* are: L, a and b; and *x_s_* is ΔE.

Cookie samples’ storage stability:

(12) $$S_{7i}=\frac{\sum_{v=1}^{2}\left(\frac{x_{vi}-x_{vmin}}{x_{vmax}-x_{vmin}}\right)}{2}$$

Total quality cookie samples’ Z-score:

(13) $$S_{i}=\left(0.15\cdot {S_{1}}_{i}+0.15\cdot {S_{2}}_{i}+0.15\cdot {S_{3}}_{i}+0.15\cdot {S_{4}}_{i}+0.15\cdot {S_{5}}_{i}+0.15\cdot {S_{6}}_{i}+0.1\cdot {S_{7}}_{i}\right)\cdot 100\%$$

where cookie samples’ nutritive (*S*_1*i*_ to *S*_3*i*_) and technical (*S*_4*i*_ to *S*_6*i*_) quality characteristic Z-score values contributed with equal 45% significance to every quality group, while storage stability quality characteristic Z-score values (*S*_7*i*_) contributed with 10% significance to the total Z-score.

max [Si]→optimum(14)

Microsoft Excel ver. 2016 (Microsoft Corporation, Redmond, WA, USA) was used for the Z-score values calculation.

## 3. Results and Discussion

### 3.1. Chemical and Mineral Matter Content

The natural habitat of black goji berries is usually arid and semi-arid regions of Central Asia [2], hence analyzing chemical and mineral matter content in black goji berries grown in Serbia is the first step in investigating its potential and application. In Table 1, the results of chemical and mineral matter content of LBGBP are presented. The low moisture content after lyophilization is beneficial to extend the shelf life.

Protein content indicates that LBGBP can contribute to the protein fraction in food formulations to some extent, although they should not be considered a primary protein source. The sugar content and total carbohydrate content are expectedly high, typical for plant-derived by-products, reflecting the presence of residual glucose and fructose, which may contribute to sweetness and browning reactions during baking, while also serving as an energy source and functional component in food systems. Cellulose content indicates a significant amount of insoluble dietary fiber, known to improve digestive health and enhance texture in bakery products, together with relatively high ash content, suggesting considerable mineral matter. The obtained fat content is low, making LBGBP a low-lipid ingredient suitable for low-fat formulations. The obtained results of all tested LBGBP chemical content responses are in accordance with the literature data for black goji berries grown in other regions of the world [1,2].

As suggested by the ash content results, LBGBP is a rich source of K, Mg, Ca and Fe, while Zn is present at a moderate level [26].

The cookie product is a complex, multi-component system consisting of macromolecules such as proteins, carbohydrates, lipids, and additives [27].

The incorporation of LBGBP as a partial substitute for wheat flour significantly influenced both the chemical composition and mineral content of the cookies (Table 1). A clear trend was observed with increasing substitution levels (0–10%), reflecting the compositional characteristics of the added raw material.

The cookies’ moisture content statistically significantly increased with the increase in the LBGBP flour substitution, where the maximal level of substitution (10%, C10) increased the cookies’ moisture content by 16.22%. The substitution of wheat flour with a matrix (LBGBP) rich in dietary fiber, polysaccharides, and anthocyanins in a lyophilized form, all of which exhibit strong water-binding properties, consequently led to higher water retention, reduced water mobility and altered dough hydration behavior [2,28]. Although the LBGBP increased the moisture content of samples, they were still below 5%, recommended for the products to stay stable during the storage and self-life period [27], while also indicating that water is predominantly in a bound form, thus increasing product stability.

The control cookie sample is characterized by a low protein content (6.21% d.m.), as well as high fat (12.26% d.m.) and sugar (56.69% d.m.) contents. The cookies’ protein content showed a statistically significant increase with the increase in LBGBP flour substitution (up to 11.59% for the maximal substitution, C10), as a result of LBGBP being relatively rich in plant-based proteins (Table 1), including essential amino acids such as lysine, arginine, and leucine [2,28]. However, this increase is accompanied by the dilution of gluten-forming proteins, which has implications for the dough structure. In contrast, fat, sugars, and total carbohydrate content declined with LBGBP flour substitution, in the case of fat and sugars statistically significantly (7.42% and 7.21% in the case of samples with the maximal level of substitution, respectively), while in the case of total carbohydrate response, it was statistically insignificant (1.39% in the case of the sample with the maximal level of substitution, C10). The obtained results can be explained by the chemical composition of LBGBP (Table 1, [28]), where the addition of this raw material in cookie recipes reduced overall fat concentration in the final product and increased water-holding capacity and fiber content, possibly limiting fat migration and oil absorption during baking [29], and also limiting available reducing sugars, as part of the mass transfer to non-digestible carbohydrates [2] or partial involvement in Maillard reactions.

The incorporation of LBGBP as a partial flour substitute resulted in a statistically significant increase in both cellulose and ash content responses in the cookie samples. Cellulose content in samples with the maximal LBGBP flour substitution showcased a 3.33-fold increase, while ash content in the same cookie sample was increased by 53.72%, as a direct consequence of the integration of cellulose and ash-rich raw material [2,28] into the cookie matrix. This increase introduces a discontinuous phase within the dough matrix, disrupting the gluten–starch network and affecting the mechanical and hydration properties in a negative manner.

The daily requirements for mineral content are small but essential for the normal functioning of the body. For this reason, it is desirable to enrich cookies with these compounds to prevent changes in metabolism or the prevention of certain disease development [30,31].

Table 1 also shows the results of the cookie samples’ mineral matter content. On the basis of the control cookie sample, C0’s, mineral matter content result, it can be considered a mineral matter-scarce product, lacking in overall mineral density and containing minimal levels of essential macro- and trace elements [32].

On the other hand, goji berries are not only valued for their bioactive compounds but also for their notable content of essential minerals, which contribute to their growing reputation as a functional food component [33]. In this present study, the authors analyzed the mineral composition of freeze-dried goji berries and observed the presence of microelements relevant to human nutrition, required for normal physiological function and overall health. The LBGBP inclusion in the cookies’ formulation led to a statistically significant increase in the following mineral elements: Zn, Fe, K, Mg, Ca and Mn. The 10% substitution of flour with LBGBP (sample no. C10) increased the cookies’ content of these minerals by 20.28%, 19.49%, 3.04 times, 64.87%, 56.10%, and 22.47%, respectively. Potassium (K) was identified as the most abundant mineral in goji berries, consistent with the previously reported literature [34]. This high potassium content contributes to the fruit’s potential in supporting cardiovascular health, particularly in blood pressure regulation. Calcium and magnesium were also present in significant amounts, which aligns with findings from studies by Donno et al. [35] and Qian et al. [5] and reflects the berries’ potential contribution to bone metabolism and neuromuscular function. Increases in the content of all these minerals in cookies may lead to improved immune function and enzyme activity (Zn) [36]; oxygen transport and cognitive function (Fe) [37]; fluid balance, nerve function and muscle contraction (K) [28]; bone health and muscle relaxation (Mg and Ca) [38]; as well as bone formation, antioxidant function and wound healing (Mn) [39]. High iron content in goji berries is notable compared with other fruits, making them a potential dietary source for populations at risk of iron deficiency. This is consistent with results from Wang et al. [40].

All cookie samples demonstrated Cu levels below the level of detection, while the cookies’ Na content statistically significantly decreased with flour substitution with LBGBP, which can also be significant to sodium-sensitive consumers. One serving (30 g) of C10 cookies would provide 1.98% and 4.53% of the dietary reference intakes for K and Fe, respectively.

### 3.2. Total Phenolics and Total Anthocyanin Content

Lyophilization is chosen as the dehydrating process due to its premium role in the stabilization and preservation of black goji berries’ polyphenol-rich profile and structural integrity by minimizing thermal degradation and oxidative losses, regardless of the process’s high energy demands and overall costs [41].

LBGBP demonstrated high levels of both TPC and TAC (Supplementary Materials, Table S2), especially when compared to red goji berries (*L*. *barbarum*) and other common fruits [1,3,17]. In our analysis, LBGBP showed a TPC of 2.37 g gallic acid equivalents (GAE) per 100 g and TAC of 1.61 g per 100 g (cyanidin-3-glucoside equivalents). This phenolic richness is consistent with the literature reports that state black goji far exceeds red goji in these phytochemicals, and can be attributed to the plant’s adaptation to environmental stress, where phenolic compounds serve as protective agents against UV radiation and oxidative stress [42,43]. Islam et al. [3] found that the average content of phenolics in black goji berries was 8.33 mg GAE/g dry weight, about 2.6-fold higher than the phenolic content in red goji (3.16 mg GAE/g). In the same study, the anthocyanin content of black goji was significantly higher than that of red goji, which is a well-known source of carotenoids (e.g., zeaxanthin). Anthocyanins are the most abundant group of phenolics present in black goji berries—in tested LBGBP samples they comprise more than 50% of TPC (1.61% of dry weight), which is significantly higher than the literature data for black goji berries. Their high concentration is related to their biological role as pigments and antioxidants. Furthermore, the structural characteristics of black goji anthocyanins—particularly the prevalence of acylated glycosides—contribute to their relatively enhanced stability compared to non-acylated forms, due to intramolecular co-pigmentation effects that protect the flavylium core from degradation [3,43]. TAC determined by Tang et al. [43] in black goji berries dried at 52 °C for 6h by the pH differential method was almost two times less compared to the TAC of LBGBP (845.6 ± 43.9 mg C3G/100 g DW). Also, compared to other valuable natural sources of anthocyanins such as bilberry, raspberry, blackcurrant, or chokeberry, a several-fold-higher TAC was found in our samples [21,44,45]. TAC in dry chokeberries, which are considered one of the richest sources of anthocyanins, extracted under different conditions was in the range of 0.21–0.27% DW according to Ćujić et al. [46]—that is significantly less than the obtained results for LBGBP. Although various factors such as geographic and environmental ones, and extraction conditions [47], could influence TPC and TAC, the obtained results underline black goji berries as a rich source of these bioactive compounds.

With the increase in the percentage of white wheat flour substitution with LBGBP (0–10%) in cookies, TPC and TAC statistically significantly increased to the levels of 58.09 mg GAE/100 g and 10.12 mg CGE/100 g of cookies, respectively (Supplementary Material, Table S2). This increase reflects a direct enrichment effect, as LBGBP serves as the primary source of phenolic compounds in the formulation. As was expected, the highest content of both TP and TA was determined in the cookies where the highest share of the flour was substituted with LBGBP (sample C10). It was noticed that the cookies’ production had a higher influence on TAC, which showed a more pronounced decrease than TPC. According to the formulation, the expected values for TPC in the cookies, calculated on the basis of incorporated LBGBP quantities, were between 1.6 and 1.8 times higher than those experimentally obtained, and 7.2–23 times higher for TAC. This decline in the content of active compounds could be expected, due to several reasons. Firstly, anthocyanins are highly sensitive to elevated temperatures encountered during baking. Thermal degradation leads to cleavage of glycosidic bonds, structural transformation of the flavylium cation into less stable forms, and subsequent polymerization or breakdown into phenolic acids and other degradation products. Secondly, oxidative degradation plays a major role, as exposure to oxygen during mixing and baking promotes the formation of reactive oxygen species that can attack phenolic structures, particularly anthocyanins due to their conjugated double-bond system. Thirdly, interactions with the food matrix significantly affect the measurable content of phenolics. Phenolic compounds can form non-covalent interactions (hydrogen bonding, hydrophobic interactions) with proteins and polysaccharides, reducing their extractability and thus their apparent concentration during analytical determination. Additionally, the pH conditions within the cookie matrix may shift during baking, influencing anthocyanin stability. At higher pH values, anthocyanins are converted from the stable flavylium cation form into less stable quinoidal bases and chalcone structures, which are more prone to degradation [48].

### 3.3. Results of the LC/MS Analysis

The samples LBGBP, C0, and C10 were analyzed using an untargeted approach based on Orbitrap LC-HRMS. A data-dependent acquisition (DDA) in both positive and negative modes was employed to selectively fragment the most intense ions, producing high-quality MS/MS spectra to improve metabolite identification [49]. Feature extraction and peak alignment were executed using techniques incorporated in MS-DIAL, an open-source LC-MS data processing software [50]. To limit false-positive identification of metabolite features, the extraction sensitivity was diminished by establishing a high minimum peak height threshold. Additionally, all extracted ion chromatograms were visually assessed to verify Gaussian-like peak forms, thereby ensuring the veracity of the identified features. A ten-fold sample average-to-blank average change filter was additionally employed to further diminish false-positive detections. Compound annotation was executed with chemical formulas derived from accurate mass, isotope ratios, and MS/MS fragments sourced from databases incorporated into the MS-FINDER software, version 3.61 (Supplementary Materials, Table S3). The MS data of structural candidates obtained by MS-FINDER, in conjunction with the UV spectra acquired, were compared to those documented in the literature [45]. The eight main metabolites including five anthocyanins (A1–A5), phenolic acid (5-*O*-CA) and two spermidine derivatives (S1, S2) found in this study are shown in Table 2.

These results are in accordance with previously published findings where anthocyanins are also identified as the main group of active compounds, besides phenolic acids, alkaloids and other groups of nutritional and functional components detected in black goji fruit [47,51]. It was also confirmed that petunidin derivatives are the dominant anthocyanins, besides the derivative of malvidin (A4), mainly present in the acetylated 3,5-diglycoside pattern that is characteristic of black goji fruit [52]. In comparison to other anthocyanin-rich fruits, black goji’s profile is unique. Berries like bilberry, blackberry, and blackcurrant contain mostly non-acylated anthocyanins (e.g., simple 3-*O*-glucosides of delphinidin, cyanidin, malvidin, etc.) [3]. In LBGBP only 5-*O*-CA was identified as a representative of phenolic acids. Hydroxycinnamic acid derivatives are usually identified in goji fruits, 3-*O* or 5-*O* caffeoylchinic acid isomers being dominant, followed by caffeic and ferulic acid in some specimens [52,53]. From the group of phenylamides, two spermidine derivatives (S1 and S2) were identified in LBGBP. Spermidine and spermine are the only natural polyamines widely distributed in living organisms including plants [54]. They are usually present in conjugation with caffeoyl and/or dihydrocaffeoyl and also glycosilation occurs often [47]. Polyamines play an important role in the normal function of DNA, RNA, proteins and other macromolecules. They are involved in cell growth and proliferation but also in apoptosis, autophagy, the antioxidant mechanism and immune regulation. Even though cells are able to produce polyamines by themselves, it has been confirmed that dietary intake of these compounds could have positive effects on human health especially a general anti-aging effect and improving the function of the brain, liver, kidney, cardiovascular and immune system [53,55]. Besides whole-grain products, vegetables, and legumes which are known as the highest spermidine-containing plant sources, goji berries have recently attracted attention as a promising source of phenylamides [7,53].

Our results showed that the investigated compounds, found only in LBGBP and not in the C0 samples, remained detectable in the C10 cookie samples, indicating that manufacturing and baking procedures did not inactivate the detected compounds, and confirming that the cookie samples with LBGBP flour substitution had improved functional compound composition. These results can be explained by the obtained balance between degradation mechanisms (thermal breakdown, oxidation) and stabilizing factors (protective effects of the food matrix, structural stability of acylated anthocyanins and possible interactions with proteins and polysaccharides that reduce reactivity) providing the combined effect of molecular structure and matrix-induced protection, rather than simple resistance to processing.

### 3.4. Results of the HPLC Analysis

Quantification of dominant phenolic compounds 5-*O*-CA and A1–A3 in LBGBP and cookies was performed by HPLC-DAD analysis. The obtained results for cookies are shown in Table 3.

The most dominant anthocyanin detected in LBGBP was A3, which reached 165.5 ± 5.89 mg/100 g and accounted for approximately 75% of the total anthocyanin content. Among the other quantified compounds, 5-*O*-CA was also present at a relatively high level (49.4 ± 1.19 mg/100 g), followed by A1 (40.4 ± 1.08 mg/100 g). In contrast, the remaining petunidin derivatives, A2 and A5, and the malvidin glucoside A4 were detected at substantially lower concentrations, namely 10.2 ± 0.18, 1.7 ± 0.03 and 1.9 ± 0.03 mg/100 g, respectively. These results indicate that LBGBP is characterized by the predominance of a highly abundant acylated petunidin 3,5-diglycoside, accompanied by smaller amounts of other anthocyanins and phenolic acid. This compositional profile is in accordance with the typical anthocyanin profile reported for black goji and suggests that A3 is the principal compound contributing to the intense purple–blue coloration and the high total anthocyanin content of black goji [56]. In addition, the relatively high level of 5-*O*-CA further supports the potential antioxidant and antidiabetic activity of LBGBP.

Anthocyanins A1–A3, together with 5-*O*-CA, were detected in the fortified cookies C2.5–C10, whereas they were absent from the control formulation (C0). This confirms that LBGBP was the sole source of these bioactive compounds in the final products. Among the enriched samples, 5-*O*-CA (1.12–4.85 mg/100 g) and A3 (0.93–4.51 mg/100 g) were consistently the most abundant individual phenolic compounds. Their concentrations increased significantly with increasing levels of LBGBP flour substitution, indicating a dose-dependent incorporation of the main LBGBP phenolics into the cookie matrix. This trend demonstrates that higher LBGBP addition effectively enhances the content of structurally relevant anthocyanins and chlorogenic acid derivative in the final product. Together with the results for total anthocyanin content, antioxidant activity and antidiabetic potential, these findings support that the compositional dominance and thermal stability of A3 and 5-*O*-CA are key factors underlying the functional improvement observed for LBGBP-enriched cookies as explained in the following sections.

Storage temperature statistically significantly affected phenolic stability. Samples kept at 4 °C for a 30-day period generally retained higher levels than those stored at 25 °C or in a stability chamber at 40 °C, underlining the thermal and oxidative sensitivity of these compounds. Notably, anthocyanin degradation was more pronounced than that of 5-*O*-CA, consistent with the literature on the susceptibility of anthocyanins to pH, temperature, and light [56]. The obtained result indicates that increasing LBGBP substitution markedly enhanced the phenolic profile of cookies, with refrigerated storage being the most effective at preserving these functional compounds. Although refrigerated storage resulted in the highest retention of phenolic and anthocyanin compounds, such conditions are not representative for typical cookies with commercial storage practices. Therefore, the results obtained at 4 °C should be interpreted as indicative of the intrinsic stability limits of LBGBP-derived bioactive compounds rather than as a direct recommendation for conventional retail storage. The observed stability at room temperature (25 °C) however demonstrates that LBGBP-enriched cookies retain a substantial proportion of supplemented functional compounds under usual commercial conditions.

### 3.5. Technical Quality Characteristics

Substituting wheat flour with any alternative ingredient in the fundamental cookie’s formulation can significantly influence technical qualities, such as shape and texture. The mechanical mixing process affects the ingredient’s interaction, creating a distinct shape, consistency, appearance, color, and flavor during the final baking phase [12,57].

In Table 4, the results of the cookies without and with LBGBP flour substitution in technical quality testing are presented. The first two presented parameters of BWL and DWL are crucial indicators of the technical quality of the cookies because they reflect the final texture and yield of the cookie product [57].

The addition of different shares of LBGBP in the cookie formulation statistically significantly affected the decrease in both *BWL* and *DWL* responses. A statistically significant reduction in these parameters was already observed at the LBGBP substitution level of 5% and 2.5%, compared to the control sample (C0), for *BWL* and *DWL*, respectively, while the highest substitution level (10%—sample no. C10) lowered *BWL* and *DWL* by 36.75% and 75.58%, respectively. These results are in accordance with the research of Nićetin et al. [25] and Filipović et al. [12], where a decrease in cookies’ *BWL* and *DWL* responses was observed with the addition of dehydrated celery and dehydrated peaches, respectively.

LBGBP has a high content of cellulose (Table 1), leading to higher water absorption and weakening of the protein network. Cellulose fibers can bind a certain amount of water, which leads to better moisture distribution in the dough. As the temperature increases during baking, the water binds more strongly and evaporates less [25]. Similar effects have been found for high-fiber or polyphenol-rich ingredients that retain more moisture content during thermal processing [58]. Additionally, the dense structure and increased moisture retention originating from LBGBP may contribute to lowered mass loss. This combination of physical entrapment and molecular interactions reduces moisture migration and vapor diffusion, thereby lowering mass loss during thermal processing. The observed decrease in *BWL* and *DWL* with increasing LBGBP levels is therefore not only a compositional effect but also a consequence of altered water mobility and the matrix structure.

The physical attributes (thickness, average diameter, and diameter-to-thickness ratio) of cookies are important for technical quality control and decision making around the packaging material and package design [59]. The substitution of flour with LBGBP in amounts ranging from 2.5% to 10% resulted in a statistically significant reduction in the thickness of the cookies compared to the control sample C0, while the change in R response was not profound. The maximal level of substitution resulted in the cookies’ T response decreasing by 13.01% and R response increasing by 5.38%.

The diameter-to-thickness ratio, - R/T, statistically significantly increased with the increasing level of flour substitution, indicating the cookie’s shape deformation with the LBGBP incorporation into the cookies’ formulation, to the maximal extent of 25% (sample no. C10 in comparison to sample no. C0).

The incorporation of LBGBP into the cookie’s formulation affects the destabilization of the dough structure, in agreement with Wang et al. [60] and Nićetin et al. [25], due to reduced gluten content and altered dough viscosity originating from LBGBP containing no gluten-forming proteins, which promote spreading during baking, due to a weaker dough structure. From a mechanistic standpoint, the increased shape deformation can also be linked to competition for water between fiber and gluten proteins. Fiber components in LBGBP bind water preferentially, limiting gluten hydration and preventing proper network development. Consequently, the dough exhibits lower cohesiveness and higher plasticity, promoting spreading during baking.

In Table 4, textural analysis of cookies without and with LBGBP flour substitution, defined by the parameters of hardness and fracturability, is also presented. These parameters are important for assessing the cookies’ technical quality, where the force required to cause the cookies’ breakage represents its hardness, and the fracturability represents the fragility of the texture and its crumbliness [61].

The incorporation of the LBGBP into the cookies’ structure led to statistically significant changes in both tested instrumental textural responses, where hardness declined while fracturability increased with increasing levels of flour substitution. Sample no. C10, with 10% LBGBP flour substitution, was characterized by only 35.30% of the hardness and by 4.24-times-higher fracturability than the control sample (C0), indicating a significant technical quality decline and potential practical problems. This phenomenon can be further explained by reduced starch gelatinization efficiency, as fiber competes for available water, limiting starch swelling and gel formation. The resulting matrix is less continuous and more prone to mechanical failure. While such changes may negatively affect technical quality from an industrial standpoint, they are consistent with the behavior of fiber-enriched bakery systems. Further optimization from the aspect of texture quality for industrial commercialization is needed.

The obtained instrumental textural responses can be explained by the dilution of the original gluten network, increased dietary fiber content, and moisture retention, all contributed by LBGBP incorporation in the cookies’ structure, disrupting the protein–starch matrix and resulting in a less compact structure. These proposed hypotheses are consistent with previous research indicating similar softening effects upon inclusion of fruit powders or fiber-rich materials in baked goods [57].

The color of the products is one of the most influential factors in customer acceptability, due to its effect on consumer perception and acceptability [62].

Table 4 also presents the four instrumental color responses for the cookie samples without and with LBGBP flour substitution. The substitution of flour with increasing LBGBP quantities in the cookie recipe led to a statistically significant change in all cookies’ instrumental color responses, where L*, a*, and b* values decreased and ∆E values increased. The addition of LBGBP to the cookies’ formulation caused a shift from a red to a green tone, and from a yellow to a blue tone, yielding an overall darker color. The highest LBGBP substitution level (10%, C10) led to lightness decreasing by 35.59 percentile points, while total color difference reached the level of 38.40, in comparison with the control cookie (sample C0). These trends are also visually noticeable from Figure S1.

The obtained results demonstrate that LBGBP, being richly pigmented due to anthocyanins and other polyphenolic compounds (Table S2), significantly influenced and reduced the cookies’ lightness, as previously reported for other purple or red fruit powders [63].

### 3.6. Biological Activity Testing

#### 3.6.1. Results of the DPPH Radical Scavenging Activity

LBGBP and cookies C0 and C10 were selected for activity testing, since C10 exhibited the highest content of phenolic compounds and thus represents the maximum expected biological effect. Consequently, comparison between the C0 and C10 samples could provide maximal insight into the LBGBP’s contribution. The results of radical scavenging activity testing using the DPPH assay are presented in Table 5.

Among samples, LBGBP demonstrated the strongest antioxidant activity, with a significantly low IC_50_ value of 4.01 ± 0.04 mg/mL. In contrast, C10 exhibited weaker activity with an IC_50_ of 149.1 ± 0.6 mg/mL, suggesting a substantial reduction in antioxidant potential, which aligns with the concentration of LBGBP present in the cookies and with the cookies’ manufacturing and baking procedures. The control sample C0 did not show measurable activity under the test conditions, indicating an absence of antioxidative activity and scarce functional qualities of the basic cookie formulation. Benefits of LBGBP and C10 are primarily attributed to the bioactive compounds present, particularly anthocyanins and chlorogenic acid derivatives, which, according to the literature, exhibit strong antioxidant properties [64].

The presence of high antioxidant levels in LBGBP, along with the enhanced antioxidant activity observed in cookies enriched with goji extract, suggests that LBGBP holds promise as a functional ingredient for enhancing the quality of baked goods. Numerous studies have reported comparable findings, highlighting the nutritional improvements in bakery items, such as cookies and biscuits, when fortified with various types of polyphenol-rich fruits. According to Raczkowska et al. [65], enriching shortbread cookies with ingredients such as blackcurrant pomace can enhance their antioxidant properties. Cookies enriched with 9% quince powder showed greater radical scavenging activity than those with the same amount of freeze-dried Japanese quince, likely due to polyphenol enrichment and the formation of Maillard reaction compounds [66]. Given the naturally low polyphenol content of wheat flour [67], incorporating LBGBP can significantly boost the nutritional profile of such products. Additionally, increasing the concentration of natural antioxidants in bakery products could probably help in extending their shelf life by reducing fat oxidation, a key factor influencing product quality.

#### 3.6.2. Antidiabetic Activity—Inhibition of α-Glucosidase and α-Amylase

Inhibition of α-Amylase and α-glucosidase is commonly used as an indicator of the potential to attenuate postprandial glucose levels. Therefore, cookies exhibiting such activity may contribute to improved glycemic control [68,69].

As shown in Table 5, both LBGBP and C10 cookies inhibited α-glucosidase activity. The IC_50_ values showed that LBGBP was more effective (1.65 mg/mL) compared to C10 (146.89 mg/mL), which aligns with the concentration of LBGBP present in the cookies and thermal treatment of the baking operation. A similar trend was observed for α-Amylase inhibition. LBGBP and C10 exhibited inhibitory activities of 1.8718 mg/mL and 161.80 mg/mL, respectively, highlighting the effectiveness in modulating carbohydrate-hydrolyzing enzymes compared to the control cookie. In a clinical study, Cai et al. [70], demonstrated that *L. barbarum* extract has significant potential as an adjunct treatment for type 2 diabetes by improving blood glucose control and lipid profiles.

This dual inhibition gives LBGBP a synergistic effect in moderating blood sugar spikes, offering comprehensive support for glycemic control. Although the basic cookie formulation used in this research was a universal one, it could indicate a suitable alternative for individuals with diabetes or obesity, lifestyle-related conditions which often require dietary restrictions, particularly regarding sweet snacks. Incorporating LBGBP into similar modified products without sugar could offer a promising way to diversify snack options for these individuals without compromising their dietary needs, providing new types of functional food.

### 3.7. Results of the Storage Stability

All cookies formulations underwent stability testing for 30 days under different storage conditions, protected from day light and kept at 4, 25, and 40 °C. TPC, TAC, and the content of the dominant phenolic compounds was monitored (Table 6).

The presence of LBGBP significantly increased TPC and TAC in enriched cookies (C2.5–C10) compared to the control (C0) throughout the 30 days of storage under all tested conditions. The control sample showed consistently low TPC (5.10–5.54 mg GAE/100 g) and no detectable anthocyanins, while enriched formulations exhibited markedly higher initial values. For example, at 25 °C, C10 reached 43.52 ± 2.08 mg GAE/100 g and 8.02 ± 0.20 mg/100 g TAC.

These findings align with previous studies reporting the high TPC and TAC of *L*. *barbarum* berries and their potential as functional ingredients in bakery products [71,72].

Storage led to a general decline in phenolic compounds, with the most pronounced losses observed at 40 °C. In C10, the content of individual phenolics decreased by more than 50% under these conditions, whereas TPC remained relatively stable, with a statistically significant decrease only at 40 °C (39.91 ± 0.44 mg GAE/100 g). In C2.5, only 5-*O*-CA and A3 were detected after storage, both at substantially lower levels compared to fresh samples.

Anthocyanins were more sensitive to storage conditions, particularly at elevated temperature. In C10, TAC decreased to 4.54 ± 0.11 mg/100 g at 40 °C, with a similar >50% reduction observed for individual anthocyanins. This degradation pattern is consistent with many previous studies indicating that anthocyanins are thermolabile and highly sensitive to heat and oxidation during storage [73,74]. Nevertheless, anthocyanins with an acetylated structure are shown to be more stable over time and under higher temperatures [52].

The obtained results indicate that LBGBP contributes to enhanced retention of bioactive compounds in cookies, although temperature remains a critical factor affecting their stability.

### 3.8. Results of the In Vitro Release Study

The in vitro release analysis of C10 was performed at pH 1.2 and pH 6.8 to simulate gastric and intestinal environments, respectively, in order to assess the pH-dependent release behavior and potential bioaccessibility of bioactive compounds during gastrointestinal transit from cookies fortified with LGBGP [23]. Anthocyanins (A1 and A3) and 5-*O*-CA were selected as markers, as they were the dominant bioactive constituents in LGBGP.

The release profile of anthocyanin compounds demonstrated a clear pH-dependent behavior, with significantly higher dissolution observed at pH 1.2, where nearly 100% release was achieved within 60 min (Supplementary Materials, Figure S2). In contrast, at pH 6.8, the release was markedly lower, reaching only about 50% after 120 min, suggesting greater anthocyanin stability and solubility under acidic conditions (Supplementary Materials, Figure S2). Anthocyanin stability and structural integrity are influenced by the pH conditions of the gastrointestinal environment, as well as by the number of hydroxyl groups present in the molecule [75]. A similar trend was reported by Ponjavić et al. [76]. They utilized lignocellulose from wood waste to produce bacterial nanocellulose which was further functionalized through simple soaking adsorption of the black raspberry pomace extract. An in vitro release study was conducted with cyanidin-3-*O*-rutinoside as the marker compound where higher content was detected in pH 1.2 than in pH 6.8.

In the case of 5-*O*-CA, the release profile differed slightly, with approximately 80% release observed at pH 6.8, compared to nearly complete release (~100%) at pH 1.2 under identical experimental conditions. Our data were consistent with previous findings. Namely, Narita and Inouye [77] showed that the stability of 5-*O*-CA was shown to decrease in a pH-dependent manner, with greater stability observed under more acidic conditions. At low pH (acidic), the molecule of 5-*O*-CA remains mostly in its protonated (non-ionized) form, which often has higher solubility in aqueous environments. Phenolics primarily interact with food macromolecules through reversible non-covalent forces (e.g., hydrogen bonds, hydrophobic and van der Waals interactions). These interactions are influenced by factors such as pH, temperature, ionic strength, and the structural properties of both phenolics and the food matrix [78]. Acidic conditions can cause partial breakdown or swelling of the food matrix, enhancing the release of bound compounds like neochlorogenic acid and anthocyanins.

The high release of anthocyanins under gastric conditions suggests that a substantial fraction becomes bioaccessible in the stomach; however, their stability decreases at neutral-to-slightly alkaline pH, which may limit absorption in the small intestine [79]. Although known for their inherent bioavailability [80], partial absorption can occur in both the stomach and small intestine, while a significant proportion reaches the colon, where they undergo microbial biotransformation into potentially bioactive metabolites [79,81]. Therefore, despite reduced release at intestinal pH, anthocyanins from LBGBP-fortified cookies may still contribute to systemic and local health effects through a combination of direct absorption and colonic metabolism.

### 3.9. Evaluation of the Overall Quality

The cookies’ quality assessment and multi-criteria optimization was performed by using Z-score analysis, combining nutritive, technical and storage stability parameters into a single, integral, dimensionless score (Table 7).

Nutritive scores (*S*_1_–*S*_3_) increased consistently with increasing levels of LBGBP substitution, where the highest Z-score values were observed at 10% substitution.

Storage stability scores (*S*_7_) also improved with LBGBP addition, indicating the antioxidant and antimicrobial effects of polyphenols, which help preserve bioactive compounds and delay degradation during storage [82].

Technical quality Z-score values (*S*_4_–*S*_6_), however, declined at higher LBGBP flour substitution levels, indicating the cookie samples’ compromised structure and mechanical properties.

The highest total Z-score values for the samples with LBGBP substitution were obtained for sample C10 (65.96%), indicating a stronger effect of nutritive enhancement than technological quality decline.

## 4. Conclusions

Black goji grown and processed in Serbia showed high phenolic and anthocyanin content, along with a favorable mineral profile, comparable to berries from their native habitat. The substitution of different white flour shares with lyophilized black goji berry powder (LBGBP) in the cookies’ formulation significantly improved their overall nutritional profile by increasing protein, dietary fiber, minerals (K, Mg, Ca, Fe, Zn, Mn) and bioactive compounds, concurrently reducing fat, sugar and sodium levels. Phenolic acids, anthocyanins and spermidine derivatives originating from LBGBP were retained in cookies after manufacturing and baking, where storage for 30 days at refrigerated temperatures (4 °C) preserved the phenolic compounds in the highest quantities. Enrichment of cookies with LBGBP provided significant improvement in antioxidant and antidiabetic activity compared to the control cookie, underlining its potential as a functional ingredient. As for technical quality, the substitution of white flour with LBGBP in cookie formulation led to reduced weight loss during baking and drying and increased dough expansion, producing softer, more crumbly cookies with darker, greenish–blue hues.

The Z-score analysis was applied to optimize the formulation with the goal of functional enrichment, balanced against technical feasibility. The 10% LBGBP substitution was calculated to produce optimal overall quality, achieving the best balance between functional enhancement and technical quality. At this level, cookies showed increased bioactive compound content, antioxidant capacity, and enzyme inhibitory activity, while maintaining acceptable product characteristics.

Although the investigation was performed on a standardized cookie formulation, used as a model product, it can be concluded that LBGBP represents a promising functional ingredient for bakery products, enabling the development of cookies with improved nutritional and functional properties, at optimized substitution levels, particularly from the sensory aspect. The incorporation of LBGBP into similar modified, sugar-free products could offer a promising way to diversify snack options for individuals with diabetes or obesity which often require dietary restrictions, particularly regarding sweet snacks.

## Figures and Tables

**Table 1:** Chemical and mineral matter content of LBGBP and cookies.

	LBGBP	C0	C2.5	C5	C7.5	C10
Chemical content						
Moisture (%)	8.91 ± 0.05	4.07 ± 0.03 ^a^	4.33 ± 0.02 ^b^	4.48 ± 0.01 ^c^	4.59 ± 0.05 ^d^	4.73 ± 0.03 ^e^
Proteins (% d.m.)	12.52 ± 0.14	6.21 ± 0.06 ^a^	6.41 ± 0.05 ^ab^	6.58 ± 0.05 ^bc^	6.73 ± 0.06 ^cd^	6.93 ± 0.02 ^d^
Fat (% d.m.)	1.50 ± 0.02	12.26 ± 0.10 ^d^	12.09 ± 0.04 ^cd^	11.90 ± 0.07 ^bc^	11.73 ± 0.11 ^b^	11.34 ± 0.13 ^a^
Sugars (% d.m.)	16.87 ± 0.11	56.69 ± 0.72 ^d^	55.29 ± 0.41 ^c^	53.99 ± 0.41 ^b^	53.41 ± 0.27 ^ab^	52.60 ± 0.08 ^a^
Celulose (% d.m.)	6.64 ± 0.04	0.24 ± 0.00 ^a^	0.44 ± 0.00 ^b^	0.51 ± 0.01 ^c^	0.73 ± 0.01 ^d^	0.80 ± 0.01 ^e^
Ash (% d.m.)	7.51 ± 0.05	1.21 ± 0.02 ^a^	1.27 ± 0.01 ^a^	1.58 ± 0.01 ^b^	1.53 ± 0.00 ^b^	1.86 ± 0.01 ^c^
Total carbohydrates (%)	72.29 ± 0.74	77.05 ± 0.33 ^a^	76.85 ± 0.71 ^a^	76.70 ± 0.72 ^a^	76.61 ± 0.69 ^a^	75.98 ± 0.31 ^a^
Mineral matter content						
Zn (mg/kg)	11.82 ± 0.11	4.37 ± 0.02 ^a^	4.69 ± 0.06 ^b^	4.81 ± 0.04 ^b^	4.99 ± 0.04 ^c^	5.25 ± 0.02 ^d^
Cu (mg/kg)	<2	<2 ^a^	<2 ^a^	<2 ^a^	<2 ^a^	<2 ^a^
Fe (mg/kg)	28.03 ± 0.05	10.11 ± 0.03 ^a^	10.63 ± 0.02 ^b^	11.21 ± 0.10 ^c^	11.56 ± 0.09 ^d^	12.08 ± 0.08 ^e^
K (mg/kg)	14,542.71 ± 57.21	738.20 ± 5.36 ^a^	1131.91 ± 14.08 ^b^	1479.82 ± 13.27 ^c^	1757.94 ± 30.02 ^d^	2243.58 ± 24.76 ^e^
Mg (mg/kg)	697.76 ± 1.41	95.34 ± 0.55 ^a^	106.81 ± 0.54 ^b^	117.39 ± 1.04 ^c^	137.37 ± 1.63 ^d^	157.19 ± 1.94 ^e^
Ca (mg/kg)	1432.08 ± 10.66	224.20 ± 2.41 ^a^	233.51 ± 2.32 ^a^	252.76 ± 1.33 ^b^	301.39 ± 1.74 ^c^	349.98 ± 1.98 ^d^
Mn (mg/kg)	5.25 ± 0.04	1.78 ± 0.01 ^a^	1.82 ± 0.03 ^a^	1.92 ± 0.02 ^b^	2.09 ± 0.02 ^c^	2.18 ± 0.03 ^d^
Na (mg/kg)	2778.48 ± 11.59	3576.18 ± 25.99 ^c^	3557.51 ± 27.32 ^bc^	3527.63 ± 33.05 ^a–c^	3505.82 ± 6.90 ^ab^	3474.09 ± 29.16 ^a^

Results are presented as average value and standard deviation of three repetitions (*n* = 3). ^a–e^ Different superscript letters in the same table rows indicate statistically significant difference between observed values according to Tukey’s HSD post hoc test at *p* < 0.05.

**Table 2:** The main compounds of LBGBP and cookies identified by LC/MS analysis.

Identified Compound	LBGBP	C0	C10
5-*O*-caffeoylquinic acid (5-*O*-CA)	+	−	+
Petunidin-3-*O*-(glucosyl-*trans*-*p*-coumaroyl)-rutinoside-5-*O*-glucoside (A1)	+	−	+
N1,N10-bis(dihydrocaffeoyl)spermidine (S1)	+	−	+
N1-caffeoyl, N10-dihydrocaffeoyl spermidine (S2)	+	−	+
Petunidin-3-*O*-(caffeoyl)-rutinoside-5-*O*-glucoside (A2)	+	−	+
Petunidin-3-*O*-(*trans*-*p*-coumaroyl)-rutinoside-5-*O*-glucoside (A3)	+	−	+
Malvidin-3-*O*-(*p*-coumaroyl)-rutinoside-5-*O*-glucoside (A4)	+	−	+
Petunidin-3-*O*-(*p*-coumaroyl)-rutinoside (A5)	+	−	+

+ Identified, − Not identified.

**Table 3:** Content of dominant phenolic compounds in cookies determined by HPLC-DAD analysis (mg/100 g).

Sample	5-*O*-CA	A1	A2	A3
C0	n.d.	n.d.	n.d.	n.d.
C0 RT	n.d.	n.d.	n.d.	n.d.
C0 CH	n.d.	n.d.	n.d.	n.d.
C0 F	n.d.	n.d.	n.d.	n.d.
C2.5	1.12 ± 0.16 ^efg^	0.20 ± 0.01 ^fg^	0.14 ± 0.01 ^hi^	0.93 ± 0.06 ^e^
C2.5 RT	0.21 ± 0.02 ^i^	n.d.	n.d.	0.14 ± 0.01 ^f^
C2.5 CH	0.19 ± 0.03 ^i^	n.d.	n.d.	0.14 ± 0.01 ^f^
C2.5 F	0.29 ± 0.01 ^hi^	n.d.	n.d.	0.17 ± 0.02 ^f^
C5	2.32 ± 0.32 ^cd^	0.33 ± 0.03 ^de^	0.40 ± 0.03 ^ef^	2.14 ± 0.19 ^c^
C5 RT	0.89 ± 0.13 ^g^	0.15 ± 0.01 ^g^	0.23 ± 0.02 ^gh^	1.15 ± 0.15 ^e^
C5 CH	0.78 ± 0.07 ^efg^	0.15 ± 0.01 ^fg^	0.23 ± 0.03 ^fg^	1.10 ± 0.14 ^cde^
C5 F	1.29 ± 0.10 ^gh^	0.21 ± 0.02 ^g^	0.31 ± 0.02 ^gh^	1.60 ± 0.09 ^e^
C7.5	3.18 ± 0.36 ^b^	0.50 ± 0.05 ^b^	0.71 ± 0.06 ^bc^	3.49 ± 0.52 ^b^
C7.5 RT	0.98 ± 0.05 ^fg^	0.18 ± 0.02 ^fg^	0.31 ± 0.04 ^gf^	1.37 ± 0.11 ^de^
C7.5 CH	1.30 ± 0.14 ^efg^	0.16 ± 0.01 ^g^	0.34 ± 0.02 ^efg^	1.35 ± 0.20 ^de^
C7.5 F	1.52 ± 0.13 ^ef^	0.27 ± 0.03 ^ef^	0.48 ± 0.06 ^de^	1.97 ± 0.14 ^cd^
C10	4.85 ± 0.26 ^a^	0.65 ± 0.08 ^a^	0.87 ± 0.12 ^a^	4.51 ± 0.45 ^a^
C10 RT	2.26 ± 0.30 ^d^	0.39 ± 0.04 ^cd^	0.58 ± 0.08 ^cd^	2.91 ± 0.28 ^b^
C10 CH	1.61 ± 0.14 ^e^	0.24 ± 0.03 ^efg^	0.43 ± 0.06 ^def^	2.00 ± 0.11 ^cd^
C10 F	2.82 ± 0.21 ^bc^	0.49 ± 0.07 ^bc^	0.74 ± 0.09 ^ab^	3.42 ± 0.41 ^b^

^a–i^ Different superscript letters in the same table rows indicate statistically significant difference between observed values according to Tukey’s HSD post hoc test at *p* < 0.05; n.d.—not detected; RT—room temperature at 25 °C; CH—stability chamber at 40 °C; F—refrigerator at 4 °C.

**Table 4:** Technical quality, texture and instrumental color characteristics of cookies.

Sample No.	C0	C2.5	C5	C7.5	C10
Technical quality characteristics					
*BWL* (%)	16.98 ± 0.40 ^c^	15.58 ± 0.47 ^bc^	13.01 ± 0.68 ^ab^	12.27 ± 1.85 ^a^	10.74 ± 0.64 ^a^
*DWL* (%)	1.72 ± 0.15 ^c^	1.13 ± 0.18 ^b^	1.13 ± 0.13 ^b^	0.84 ± 0.18 ^b^	0.42 ± 0.10 ^a^
T (mm)	11.30 ± 0.15 ^c^	10.33 ± 0.11 ^b^	10.33 ± 0.08 ^b^	10.00 ± 0.12 ^a^	9.83 ± 0.08 ^a^
R (mm)	0.93 ± 0.02 ^a^	0.95 ± 0.04 ^a^	0.96 ± 0.02 ^a^	0.97 ± 0.03 ^a^	0.98 ± 0.01 ^a^
R/T	0.08 ± 0.00 ^a^	0.09 ± 0.00 ^b^	0.09 ± 0.00 ^bc^	0.10 ± 0.00 ^cd^	0.10 ± 0.00 ^d^
Texture characteristics					
Hardness (N)	14,403.72 ± 1390.29 ^b^	7316.37 ± 916.75 ^a^	7291.57 ± 996.64 ^a^	7074.50 ± 1602.9 ^a^	5084.03 ± 708.25 ^a^
Fracturability (mm)	0.63 ± 0.17 ^a^	0.68 ± 0.09 ^a^	1.44 ± 0.34 ^b^	1.62 ± 0.14 ^b^	2.67 ± 0.25 ^c^
Instrumental color characteristics					
L*	76.38 ± 0.92 ^d^	56.25 ± 2.04 ^c^	50.46 ± 0.43 ^b^	41.56 ± 0.55 ^a^	40.97 ± 0.69 ^a^
a*	2.14 ± 0.11 ^c^	−0.65 ± 0.48 ^b^	−1.17 ± 0.24 ^b^	−8.37 ± 0.08 ^a^	−8.66 ± 0.25 ^a^
b*	24.67 ± 0.50 ^e^	14.58 ± 0.28 ^d^	11.64 ± 0.27 ^c^	4.65 ± 0.34 ^b^	1.30 ± 0.20 ^a^
∆E		22.18 ± 1.01 ^a^	28.49 ± 0.56 ^b^	37.64 ± 0.47 ^c^	38.40 ± 0.40 ^c^

Results are presented as average value and standard deviation of six repetitions (*n* = 6). ^a–e^ Different superscript letters in the same table rows indicate statistically significant difference between observed values according to Tukey’s HSD post hoc test at *p* < 0.05.

**Table 5:** Results of the biological activity testing.

Sample	DPPH (IC_50_—mg/mL)	Inhibition of α-Glucosidase (IC_50_—mg/mL)	Inhibition of α-Amylase (IC_50_—mg/mL)
LBGBP	4.01 ± 0.04 ^a^	1.65 ± 0.11 ^a^	1.87 ± 0.11 ^a^
C0	n.ac.	n.ac.	n.ac.
C10	149.1 ± 2.6 ^b^	146.89 ± 0.40 ^b^	161.80 ± 4.48 ^b^
Ascorbic acid *	4.30 ± 0.05 ^a^	n.ap.	n.ap.
Acarbose *	n.ap.	142.51 ± 16.63 ^b^	22.26 ± 0.13 ^a^

^a–b^ Different superscript letters in the same table rows indicate statistically significant difference between observed values according to Tukey’s HSD post hoc test at *p* < 0.05; n.ap.—not applicable; n.ac.—not active; * µg/mL.

**Table 6:** Total phenolic content (TPC) and total anthocyanin content (TAC) after storage stability testing.

		C0	C2.5	C5	C7.5	C10
TPC *	RT	5.13 ± 0.7 ^a^	13.53 ± 0.55 ^a^	25.89 ± 0.14 ^a^	28.93 ± 0.69 ^ab^	43.54 ± 2.00 ^ab^
	F	5.35 ± 0.21 ^a^	15.96 ± 0.82 ^b^	31.56 ± 2.46 ^b^	35.02 ± 4.34 ^b^	46.63 ± 1.26 ^b^
	CH	5.54 ± 0.19 ^a^	13.05 ± 0.76 ^a^	24.13 ± 1.52 ^a^	26.11 ± 1.71 ^a^	39.86 ± 2.49 ^a^
TAC **	RT	/	1.00 ± 0.06 ^b^	3.19 ± 0.07 ^ab^	3.95 ± 0.15 ^b^	8.02 ± 0.20 ^b^
	F	/	0.96 ± 0.09 ^ab^	3.58 ± 0.15 ^b^	4.51 ± 0.18 ^c^	7.59 ± 1.08 ^b^
	CH	/	0.83 ± 0.01 ^a^	3.07 ± 0.28 ^a^	3.37 ± 0.09 ^a^	4.54 ± 0.11 ^a^

* Results for TPC are expressed as mg GAE/100 g of the samples; ** results for TAC are expressed as mg CGE/100 g of the samples; RT—room temperature at 25 °C; CH—stability chamber at 40 °C; F—refrigerator at 4 °C. ^a–c^ Different superscript letters in the same table rows indicate statistically significant difference between observed values according to Tukey’s HSD post hoc test at *p* < 0.05.

**Table 7:** Z-score values of the cookies without and with LBGBP flour substitution.

		C0	C2.5	C5	C7.5	C10
Nutritive quality score	*S*_1_	0.09	0.33	0.63	0.71	0.83
	*S*_2_	0.00	0.20	0.42	0.70	1.00
	*S*_3_	0.00	0.16	0.46	0.75	1.00
Technical quality score	*S*_4_	0.60	0.42	0.47	0.35	0.40
	*S*_5_	0.50	0.87	0.68	0.65	0.50
	*S*_6_	1.00	0.54	0.42	0.05	0.00
Storage stability score	*S*_7_	0.00	0.17	0.47	0.56	1.00
Total Score		32.91%	39.75%	50.87%	53.58%	65.96%

## Data Availability

The original contributions presented in this study are included in the article/Supplementary Material. Further inquiries can be directed to the corresponding author.

## Supplementary Materials

The following supporting information can be downloaded at: https://www.mdpi.com/article/10.3390/foods15101733/s1, Table S1: Experimental plan and formulation for cookies without and with LBGBP flour substitution; Table S2: TPC and TAC in LBGBP and cookies; Table S3: LC/MS data of identified compounds in LBGBP and cookies without and with LBGBP flour substitution (10%); Figure S1: Images of LBGBP and cookie samples (C0-C10, 0–10% of LBGBP flour substitution, respectively); (a) powered cookie samples, (b) whole cookie samples; Figure S2: Results of in vitro release analysis of cookie formulation C10 performed at pH 1.2 and pH 6.8. Legend: A1-petunidin-3-*O*-(glucosyl-*trans-p*-coumaroyl)-rutinoside-5-*O*-glucoside; A3-petunidin-3-*O*-(*trans-p*-coumaroyl)-rutinoside-5-*O*-glucoside.
