# Supplementary material for: Quality Evaluation and Multi-Criteria Optimization of Cookies Fortified with Lyophilized Black Goji
Source: Foods. 2026 May 14;15(10):1733. doi: 10.3390/foods15101733 (PMC13206468; doi:10.3390/foods15101733)
Supplement: Supplementary file 1 [file foods-15-01733-s001.zip › foods-4293291-supplementary.pdf]

SUPPLEMENTARY MATERIAL

Quality Evaluation and Multi-Criteria Optimization of Cookies Fortified with Lyophilized Black Goji

Katarina Šavikin, Gordana Zdunić, Jelena Živković, Nada Ćujić Nikolić, Dejan Gođevac, Milica Nićetin, Jelena Filipović, Vladimir Filipović

Table S1. Experimental plan and formulation for cookies without and with LBGBP flour substitution

Ingredient	C0	C2.5	C5	C7.5	C10
White wheat flour (g)	225.00	219.40	213.80	208.10	202.50
LBGBP (g d.m. / % of white flour substitution)	0 g / 0 %	5.60 g / 2.50 %	11.20 g / 5.00 %	16.80 g / 7.50 %	22.40 g / 10.00%
Margarine (g)			64.00		
Sugar (g)			152.50		
NaCl (g)			2.10		
$NaHCO_3$ (g)			2.50		
Glucose (g)			2.00		
Distilled water (g)			49.00		

Table S2. TPC and TAC in LBGBP and cookies.

Sample	TPC*	TAC**
LBGBP	2372.41 ± 0.07^{f}	1611.26 ± 0.05^{d}
C0	5.82 ± 0.57^{a}	/
C2.5	16.84 ± 2.14^{b}	1.19 ± 0.05^{a}
C5	34.92 ± 1.52^{c}	3.64 ± 0.22^{ab}
C7.5	44.58 ± 5.02^{d}	7.61 ± 0.81^{bc}
C10	58.09 ± 5.71^{e}	10.13 ± 4.53^{c}

*Results for TPC are expressed as mg GAE/100 g of the samples; **Results for TAC are expressed as mg CGE/100 g of the samples

Table S3. LC/MS data of identified compounds in LBGBP and cookies without and with LBGBP flour substitution (10%)

			NEG mode			POS mode		
No.	Metabolite name	Rt(min)	Average Mz	Adduct type	MS/MS spectrum	Average Mz	Adduct type	MS/MS spectrum
1	5-*O*-caffeoylquinic acid (5-*O*-CA)	15.7	353.08725	[M-H]-	51.15953:33875 53.18955:35157 61.90393:35028 64.91180:33416 71.01364:52309 85.02966:61103 93.03462:260141 102.38416:34926 111.04520:144008 119.80112:42019 135.04527:1026938 137.02469:184239 144.32523:40716 155.03491:213973 161.02426:345189 173.04564:3032801 179.03503:2235648 191.05614:14776635 192.06015:120196 194.55144:44185 212.11484:34382 320.96991:35443	no peak observed		

No.	Metabolite name	Rt(min)	NEG mode Average Mz	Adduct type	MS/MS spectrum	POS mode Average Mz	Adduct type	MS/MS spectrum
2	petunidin-3-*O*-(glucosyl-*trans*-*p*-coumaroyl)-rutinoside-5-*O*-glucoside (A1)	17.2	1093.30322	[M-2H]-	125.02443:1044503 126.02834:18604 137.02441:18986 139.04019:22954 145.02963:26051 147.00887:53977 148.01709:25172 149.02426:75111 151.04036:32531 163.00420:113272 163.03958:62330 165.01984:40222 167.03503:18839 175.00488:23226 177.01947:93762 189.01991:37920 191.03503:66448 192.00623:100337 193.01682:23334 203.03566:20314 205.01418:82952 205.05060:39986 206.02092:27018 207.02986:551987 217.01373:44288 219.03113:26561 226.14929:24933 227.03430:24774 231.02995:58945 232.03844:24201 233.04518:25662 243.03226:24387 247.06035:48006 255.02994:66645 256.03754:72214 259.05899:21320 270.05478:27640 271.02405:59503 271.06143:64136 272.03299:113566 273.03967:41135 275.60593:18257 281.04453:24715 282.01691:70790 283.02518:230446 284.03308:31968 285.04019:29866 287.05618:62977 289.07071:25259 296.03278:28699 297.03986:185120 298.01096:26258 298.04849:53152 299.01953:808428 299.05466:37317 300.02740:1115789 309.09784:67493 312.98822:32931 313.03543:1664514	1095.31543	[M]+	147.04359:429775 217.95103:90056 302.04105:800177 317.06445:161512192 318.06784:2297727 329.06604:87839 359.07498:332773 383.07452:148309 415.37802:94787 425.09189:155089 443.09491:179489 461.10782:288818 479.11844:652628

No.	Metabolite name	Rt(min)	NEG mode Average Mz	Adduct type	MS/MS spectrum	POS mode Average Mz	Adduct type	MS/MS spectrum
					314.04318:10866269 315.05084:19445930 316.05423:479662 317.06686:64772 324.02655:54327 325.03442:57582 327.04993:187151 328.06049:69994 329.06650:1097943 330.07043:30008 339.05142:390601 341.06586:42788 351.10675:32559 356.05466:41000 357.06119:558187 358.06522:25625 369.05945:141380 381.06125:34901 387.07309:108888 461.07080:63321 462.07889:32452 476.09586:1657937 477.10336:1800129 478.11075:56876 605.15076:32163 623.16046:686762 624.16547:35748 643.16986:137926 769.02216:30807 769.19818:406524 770.19269:24780 785.20770:26783 805.21777:191600 899.21967:31978 931.24939:373588 932.25525:42847			

No.	Metabolite name	Rt(min)	NEG mode Average Mz	Adduct type	MS/MS spectrum	POS mode Average Mz	Adduct type	MS/MS spectrum
3	N1,N10-bis(dihydrocaffeoyl)spermidine (S1)	20.0	472.24478	[M-H]-	58.02970:73405 79.82469:59001 92.89323:80812 99.73669:74421 109.02932:244633 121.02969:532327 137.06091:100975 163.04025:1384997 169.52885:79709 226.13994:77558 228.17285:460716 308.19815:10820867 309.20279:81296 334.17441:128632 350.20865:8305903 351.21146:122497 352.11133:108161 362.20935:324673 454.23898:322622 457.28287:71383 472.24542:41224944 473.24994:1069453	474.25784	[M+H]+	56.98713:428851 57.41306:459588 58.06486:596286 72.08052:3583804 88.12968:516788 100.07521:1413860 101.63602:464942 112.11144:966865 123.04338:2922803 129.13838:782760 164.06874:503565 165.05432:9369860 184.69641:495085 221.12810:1244660 222.11183:153317920 226.20866:807722 236.12767:13140337 239.13751:666988 253.15555:1131055 292.19962:873068 293.18521:5223604 294.83243:508346 310.20908:3254587 456.24667:5062778 457.23236:5892823 474.25821:52471028

No.	Metabolite name	Rt(min)	NEG mode Average Mz	Adduct type	MS/MS spectrum	POS mode Average Mz	Adduct type	MS/MS spectrum
4	N1-caffeoyl, N10-dihydrocaffeoyl spermidine (S2)	20.5	470.229	[M-H]-	121.02981:61469 135.04521:8319783 136.04819:59290 138.94397:20498 161.02444:625856 224.31387:19259 291.17157:1987728 292.79865:19376 306.18240:37737 308.19788:495015 334.17740:4382745 335.17957:57812 348.19238:33166 470.22888:345862	472.24216	[M+H]+	67.81673:183034 72.08063:4807384 100.07578:271167 112.11198:533547 123.04508:789269 129.13826:359127 163.03867:14365866 164.07065:281300 165.05415:2174553 220.09622:1985043 221.12802:426597 222.11200:64462024 223.11371:225748 234.11197:7229548 239.13869:4238218 251.13747:287202 290.18585:209910 291.16907:523631 292.20151:272150 293.18546:10853601 308.19702:723021 310.21210:11569628 322.12610:217176 336.19275:553048 348.25302:197328 454.23331:1589483 455.21704:1287203 472.24304:17447542

			NEG mode			POS mode		
No.	Metabolite name	Rt(min)	Average Mz	Adduct type	MS/MS spectrum	Average Mz	Adduct type	MS/MS spectrum
5	petunidin-3-*O*-(caffeoyl)-rutinoside-5-*O*-glucoside (A2)	21.3	947.24615	[M-2H]-	119.05038:3654 125.02420:4441 145.02945:9909 151.00359:18055 163.04028:10410 175.00330:2448 178.99850:17867 179.36705:2093 189.49379:2169 193.01384:2766 205.05078:9962 245.04568:7306 272.03571:2585 285.04196:3016 297.00400:2573 298.01248:4072 299.01987:5189 301.03430:4848 303.05103:2417 312.02750:3728 313.03149:12115 314.04340:11993 315.01187:26161 315.05402:15921 316.02280:22414 329.03049:219334 330.03836:403145 331.04599:574754 332.04974:10420 351.10953:3831 364.05640:2567 372.04999:2502 373.05511:19986 460.06293:5432 475.08536:4812 477.06760:12661 478.07712:4641 492.09103:252318 493.09888:57863 494.09430:3376 607.13165:6578 621.14044:12661 624.13544:7910 639.15656:595440 640.15747:18418 681.16217:3267 770.16626:5137 785.19366:35077 786.19861:3902 801.21063:27849 823.23535:2395 947.24139:3402	949.25854	[M]+	109.92392:6035 111.04311:4863 147.04378:88497 286.22440:4904 293.10394:5795 301.17957:6425 317.06464:204445 331.08020:6505 333.06000:93755

No.	Metabolite name	Rt(min)	NEG mode Average Mz	Adduct type	MS/MS spectrum	POS mode Average Mz	Adduct type	MS/MS spectrum
6	petunidin-3-*O*-(*trans-p*-coumaroyl)-rutinoside-5-*O*-glucoside (A3)	23.2	931.25049	[M-2H]-	124.01661:60102 125.02442:3156028<br>129.38870:50331 139.04013:108267<br>145.02963:213969 147.00888:107315<br>149.02406:207849 163.00351:299070<br>163.03990:191748 165.01891:70465<br>176.01183:52508 177.01921:114155<br>191.03496:153876 192.00671:167070<br>205.01398:86743 205.05103:154133<br>207.02991:850199 219.02982:58417<br>226.15227:85548 231.02933:116385<br>247.06073:194342 255.02933:156009<br>256.03769:320239 261.04141:72205<br>269.04434:75851 271.02582:119783<br>271.06088:199308 272.03305:208834<br>273.04285:73784 282.01712:296178<br>283.02463:537891 284.03061:119696<br>286.05011:65137 287.05591:302410<br>297.03912:356447 298.00851:61927<br>298.04855:77445 299.01941:1891470<br>299.06116:100117 300.02731:2500290<br>301.03336:60897 309.09705:114270<br>313.03528:3396446<br>314.04306:24580000<br>315.05078:44327356<br>316.05444:850103 317.06503:82270<br>324.02628:114029 325.03311:67457<br>327.05023:301083 328.06119:92541<br>329.06656:1726972 334.03226:50380<br>339.05121:597512 341.05887:58898<br>351.10809:134399 356.05298:71583	933.26239	[M]+	135.45581:428837<br>147.04349:1152522<br>224.76389:452987<br>302.04105:1804901<br>317.06439:520121536<br>318.06876:3912901<br>359.07565:1055163<br>391.94910:386817<br>443.09668:638593<br>461.10452:538526<br>479.11783:878165

No.	Metabolite name	Rt(min)	NEG mode<br>Average Mz	Adduct type	MS/MS spectrum	POS mode<br>Average Mz	Adduct type	MS/MS spectrum
					357.06082:790290 369.06339:260787 387.07251:113312 424.26138:56655 462.07928:183572 476.09540:2752114 477.10342:3375762 478.10687:128499 497.12866:124508 591.15637:49996 623.16125:3907454 624.16766:149592 769.19751:413671 874.59998:56631			

No.	Metabolite name	Rt(min)	NEG mode Average Mz	Adduct type	MS/MS spectrum	POS mode Average Mz	Adduct type	MS/MS spectrum
7	malvidin-3-*O*-(p-coumaroyl)-rutinoside-5-*O*-glucoside (A4)	25.2	945.26617	[M-2H]-	no MS/MS spectrum obtained	947.27856	[M]+	147.04382:48603 262.67227:17420 315.05078:31405 330.07275:17037 331.08020:14432717 332.08368:278212 397.08926:19887 457.83371:18017 475.12326:32621 493.13620:43663 507.60529:16715 573.31018:18457 758.54333:17224 844.69971:18972
8	petunidin-3-*O*-(p-coumaroyl)-rutinoside (A5)	29.0	769.19806	[M-2H]-	124.01612:5736 125.02447:238288 139.04024:10729 145.02994:12703 147.00920:15874 149.02466:18795 163.00388:11542 163.03998:13044 168.49925:5791 177.01909:6456 191.03566:10858 192.00606:19040 205.05075:5651 207.03009:40056 232.03676:6699 233.04599:6355 244.12068:5238 247.06268:9032 255.02936:18708 256.03851:34603 270.05396:5972 271.02359:19233 271.06158:17541 272.03275:24834 273.03928:7174 282.01724:11857 283.02457:26222 286.05035:6562 287.05685:33499 298.00998:5561 299.01968:440272 300.02710:210200	771.21002	[M]+	80.78442:12043 101.47021:11485 122.40885:10362 134.19966:10873 147.04350:48790 195.42862:10972 240.08958:12083 302.04099:96526 317.06448:14473590 318.06815:238248 717.31549:11607

No.	Metabolite name	Rt(min)	NEG mode Average Mz	Adduct type	MS/MS spectrum	POS mode Average Mz	Adduct type	MS/MS spectrum
					309.09741:6189 313.03537:20455 314.04315:2542215 315.05093:1062444 316.05545:17982 327.05124:14334 329.06680:98471 342.23541:6616 357.06094:20231 369.05972:16510 387.07071:7300 486.69775:5217 602.44495:5235 623.16205:101659 727.78845:5851			

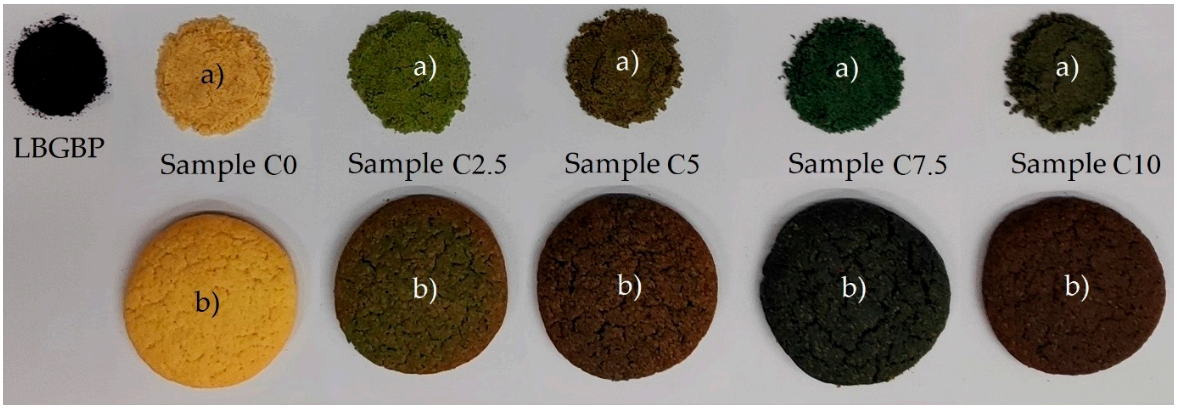

Figure S1. Images of LBGBP and cookie samples (C0-C10, 0%-10% of LBGBP flour substitution, respectively); a) Powered cookie samples, b) Whole cookie samples

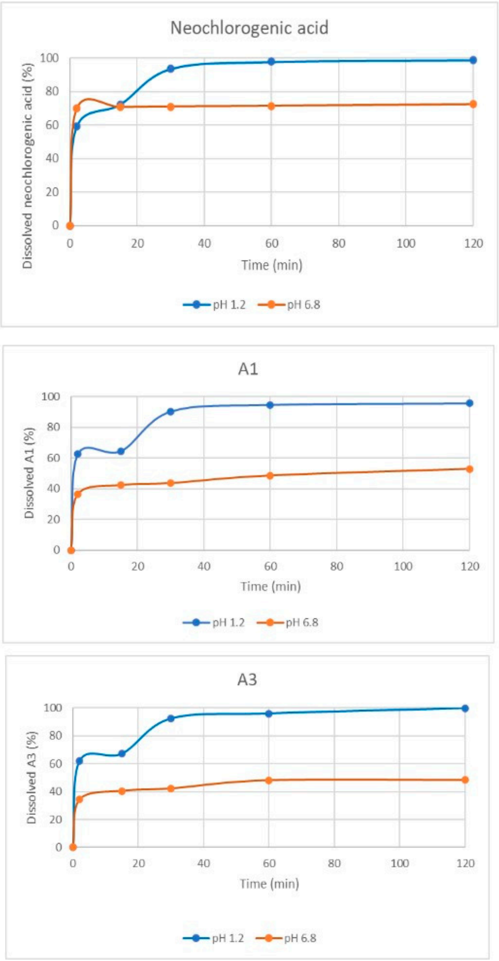

Figure S2. Results of *in vitro* release analysis of cookie formulation C10 performed at pH 1.2 and pH 6.8. Legend: A1- petunidin-3-*O*-(glucosyl-*trans*-*p*-coumaroyl)-rutinoside-5-*O*-glucoside; A3- petunidin-3-*O*-(*trans*-*p*-coumaroyl)-rutinoside-5-*O*-glucoside
